# Gut-muscle crosstalk. A perspective on influence of microbes on muscle function

**DOI:** 10.3389/fmed.2022.1065365

**Published:** 2023-01-09

**Authors:** Weixuan Chew, Yen Peng Lim, Wee Shiong Lim, Edward S. Chambers, Gary Frost, Sunny Hei Wong, Yusuf Ali

**Affiliations:** ^1^Nutrition, Metabolism and Health Programme, Lee Kong Chian School of Medicine, Nanyang Technological University Singapore, Singapore, Singapore; ^2^Centre for Microbiome Medicine, Lee Kong Chian School of Medicine, Nanyang Technological University Singapore, Singapore, Singapore; ^3^Institute of Geriatrics and Active Aging, Tan Tock Seng Hospital, Singapore, Singapore; ^4^Department of Nutrition and Dietetics, Tan Tock Seng Hospital, National Healthcare Group, Singapore, Singapore; ^5^Section for Nutrition Research, Department of Metabolism, Digestion and Reproduction, Faculty of Medicine, Imperial College London, London, United Kingdom; ^6^Division of Diabetes, Endocrinology and Metabolism, Department of Medicine, Imperial College London, London, United Kingdom; ^7^Department of Gastroenterology and Hepatology, Tan Tock Seng Hospital, National Healthcare Group, Singapore, Singapore; ^8^Singapore General Hospital, Singapore Eye Research Institute (SERI), Singapore, Singapore; ^9^Clinical Research Unit, Khoo Teck Puat Hospital, National Healthcare Group, Singapore, Singapore

**Keywords:** gut microbes, muscle function, metabolites, cytokines, sarcopenia

## Abstract

Our gastrointestinal system functions to digest and absorb ingested food, but it is also home to trillions of microbes that change across time, nutrition, lifestyle, and disease conditions. Largely commensals, these microbes are gaining prominence with regards to how they collectively affect the function of important metabolic organs, from the adipose tissues to the endocrine pancreas to the skeletal muscle. Muscle, as the biggest utilizer of ingested glucose and an important reservoir of body proteins, is intricately linked with homeostasis, and with important anabolic and catabolic functions, respectively. Herein, we provide a brief overview of how gut microbiota may influence muscle health and how various microbes may in turn be altered during certain muscle disease states. Specifically, we discuss recent experimental and clinical evidence in support for a role of gut-muscle crosstalk and include suggested underpinning molecular mechanisms that facilitate this crosstalk in health and diseased conditions. We end with a brief perspective on how exercise and pharmacological interventions may interface with the gut-muscle axis to improve muscle mass and function.

## Introduction

The gut-muscle axis describes how the gut microbiota can impact muscle mass, muscle quality and muscle function. The gut consists of trillions of microbial cells, which plays an important role in many aspects of human health and can influence muscle health through dietary fiber, proteins and metabolic by-products (1). The gut microbiota ferments non-digestible substrates such as dietary fibers to produce short chain fatty acids (SCFA) which have important regulatory functions. Emerging evidence suggests a relationship between gut microbiota and sarcopenia, which is the age-related loss of skeletal muscle mass and function. The spectrum of parameters implicated in muscle health ranges from muscle quantity which is typically measured *via* the appendicular lean mass using dual energy X-ray absorptiometry (DXA) or bioelectrical impedance analysis (BIA); muscle quality which refers to the amount of fat infiltration into muscle; and muscle function which measures the components of strength and physical performance.

Muscle health is important because skeletal muscles are major sites of insulin stimulated glucose uptake, and thus play a key role in glucose homeostasis and whole-body metabolism. Furthermore, low skeletal muscle mass is often associated with frailty in older adults which increases their susceptibility to adverse outcomes and negatively affects their quality of life. It is also associated with many metabolic diseases such as Type 2 Diabetes Mellitus (T2DM). If it turns out that the gut microbiome does indeed strongly influence muscle health *via* the gut-muscle axis, it can create new avenues of treatment to improve muscle health through direct means such as probiotics or indirectly *via* dietary interventions or prebiotic supplementation. In this article, we summarize recent animal and human studies that suggest the role of gut microbiota in influencing muscle health, and on how gut microbes may in turn be altered during certain muscle disease states. Insofar, the aim is not to provide a systematic review but rather to give a perspective based on existing evidence that exercise and pharmacological interventions provide benefit by impinging on the gut-muscle axis.

## Associations between gut microbiome and muscle

Animal studies reveal interesting insights about the gut-muscle axis. In a study on mice, it was found that germ free (GF) mice lacking in gut microbiota displayed reduced skeletal muscle weight, as compared to conventional or specific pathogen free (SPF) mice which have an intact gut microbiota and immune system (2). Upon histological examination of the tibialis anterior and gastrocnemius, fewer but larger muscle fibers could be seen. In the muscles of the GF mice, there was reduced expression of the succinate dehydrogenase (*Sdh*) gene and reduced activity of the mitochondrial SDH enzyme. The amount of mitochondrial DNA content also reduced and there was evidence of dysfunctional mitochondrial biogenesis and oxidative capacity of the soleus (oxidative) and extensor digitorium longus (glycolytic). Reduced expression of glycolytic genes was observed in these muscle groups. However, despite a possible reduction in oxidative metabolic capacity, the GF mice performed as well as SPF mice when challenged till exhaustion, suggesting the involvement of other compensatory pathways especially during the endurance phase. Importantly, upon treatment with SCFA, the muscle strength of GF mice increased as compared to the untreated GF mice, suggesting that SCFAs may be an important link between gut microbiota and muscle function. Transplantation of gut microbiota from SPF mice to GF mice helped to restore muscle mass and mitochondrial DNA content in GF mice muscle. This suggests that modulation of microbiota could potentially be used in humans as a way to treat conditions such as sarcopenia. When similar experiments were repeated in piglets, the results were largely similar to what was observed in mice (3). The GF piglets exhibited a lower growth rate as compared to control piglets with normal microbiota. A group of GF piglets was treated with fecal microbiota transplantation (FMT) from healthy adult pigs and the average body weight of piglets receiving FMT increased by ~1.4-fold compared to that of the GF piglet. Although the FMT did not completely restore growth of the GF piglet, they showed improved body conditioning and physiological traits as compared to the GF piglets. As for underpinning mechanism, the lower proportion of slow twitch muscle fibers of the GF piglets correlated with reduced SCFA contents pointing toward a role of gut microbes, specifically butyrate-producers in influencing slow-twitch muscle fiber development. In addition, the blood concentrations of triglycerides (TG), glucose and growth hormones in the FMT piglet were also significantly higher than that of the GF piglet suggesting that the introduction of gut microbes improved whole-body metabolic homeostasis in GF piglets.

In another study, when three different antibiotic regimens (1. cefoperazone, 2. enrofloxacin/ampicillin, 3. a four-drug regimen of neomycin, vancomycin, metronidazole and ampicillin) were administered to mice, there was a decrease in mass of the gastrocnemius-soleus complex of the mice (4). However, when the antibiotics were administered to GF mice, they did not lose any muscle mass as compared to the control suggesting that the effects of antibiotics on muscle mass is likely modulated through its impact on the microbiome. This could be due to the concurrent alteration of the gut microbiota, the composition of which depended on antibiotics administered. An intact gut microbiome has also been shown to be important for skeletal muscle adaptation to exercise (5). Mice treated with antibiotics to disrupt gut microbiome showed a blunted soleus and plantaris muscle fiber-type specific hypertrophy in response to progressive weighted wheel running as compared to those without antibiotics treatment. Mice which were colonized with gut bacteria through FMT from high functioning human donors had a 5.4% increase grip strength as compared to those which received FMT from low functioning human (6). While animal studies help with mechanistic underpinnings of phenotypic observations, it is also important to assess how much of these findings translate to the human setting.

It is worth noting that there are also studies the suggest negative associations between gut microbiota and whole-body lean mass. In one study when GF mice were colonized by fecal samples from age matched, conventionally raised mice, the whole-body lean mass decreased by 7–9% with a 57% increase in total body fat content. It was also associated with increased plasma leptin, fasting glucose and fasting insulin levels (7).

In another study, mice treated with pulsed antibiotic treatment (PAT) either using amoxicillin or tylosin phosphate developed larger bones with increased lean and fat mass as compared to controls. It trended toward increased bone in amoxicillin-treated mice and increased fat in tylosin-treated mice (8) and this was corroborated in antibiotic treated piglets. In tylosin phosphate-treated piglets, myofiber density and expression of genes related to type I and type IIb myofibers as well as fatty acid uptake in longissimus muscle was observed to be increased, together with gut microbe changes where the ratio of Firmicutes to Bacteroidetes was increased, while *Prevotella* and *Campylobacter* were decreased in the cecum (9).

Given its highly complex and multi-dimensional nature, the microbiota that evolve with different antibiotics and FMT regimens can exert different corresponding phenotypes. While gut microbiota does have affect muscle mass and function, these discrepant findings do suggest the need for more studies to determine the causality, functionality and directionality of the microbiota and its constituent members.

## Human studies associate gut microbe changes with metabolic- and age-related muscle loss

Patients with low muscle mass or sarcopenia, in the context of organ failure or cancer, were observed to have alterations in their gut microbiome. It was reported that patients with chronic liver disease who had lower muscle mass possessed a lower *Firmicutes*/ *Bacteroidetes* ratio than those with normal muscle mass (10). The levels of *Coprobacillus, Catenibacterium* and *Clostridium* were also lower while *Bacteroides* was higher comparing between muscle sub-groups. There was also a high relative abundance of Gram-negative bacteria and corresponding lipopolysaccharides (LPS) suggesting a possible link between gut microbes, inflammation and changes to muscle mass. There are cross-sectional human association studies that compare patients with cirrhosis-related sarcopenia with control subjects. The principal alteration in age-related sarcopenia and cirrhosis-related sarcopenia was a reduction in SCFA-producing bacteria. *Lachnospiraceae* family, consisting of *Lachnospira, Fusicatenibacter, Roseburia*, and *Lachnoclostridium*, significantly decreased in age-related sarcopenia.

Interestingly, in a study involving nursing-home residents aged 65 years or older, with increasing frailty, residents had lower levels of butyrate producing organisms, higher levels of known dysbiotic species, and higher LPS and peptidoglycan (PGN) biosynthesis. Amongst the residents, with increasing age, there was a reduction in mucin-degrading *Akkermansia muciniphila* and butyrate-producing *Ruminococcus bromii* likely due to a change in diet. With increasing malnourishment, there is increased abundance of LPS-producing *Ruminococcus gnavus* and deceased butyrate-producing *Lachnospiracae* and *Ruminococcaceae* (11). In a separate study on stool samples from frail old people, *Lactobacilli, F. prausnitzii*, and *Bacteroides / Prevotella* ratio declined sharply and *Enterobacteriaceae* increased (12). Such changes in gut microbial species may alter the inflammatory tone as *Lactobacilli, F. prausnitzii* are largely anti-inflammatory while *Enterobacteriaceae* induces pro-inflammatory effects. It has been suggested that age dependent changes in gut microbiota may be the initiator of frailty symptoms facilitated by chronic inflammation, since probiotic rescue reduces inflammation and muscle atrophy (13–15).

Admittedly, metabolic dysregulation and aging are complex conditions that encompass differences in nutritional intake, digestion and assimilation, drug use treatments (usually involving multiple drugs) and background physiology and inflammation, all of which may profoundly confound microbiome changes and muscle health. Mechanistic studies are key to elucidating how the microbiome, and its metabolites, influence muscle metabolism and survivability. Furthermore, the gut microbiota diversity may also be affected by protein intake. Briefly, in a study with professional athletes from an international rugby union squad compared against healthy male controls, there was a significant increase in gut microbiota diversity and this association also correlated with protein intake and plasma creatine kinase values (16). Greater microbiota α-diversity has been reported in athletes in associations with dietary patterns and protein consumption (16, 17).

## Involvement of gut microbiota in cancer-related muscle loss

In patients with advanced gastric cancer, cachexia was associated with intestinal barrier dysfunction (i.e., greater intestinal permeability) with a higher degree of bacterial translocation, as compared to patients with gastric cancer but without cachexia (18). Levels of Interleukin-6 (IL-6), Tumor Necrosis Factor α (TNF-α), and Interferon γ (IFNγ) correlated with bacterial translocation in patients with cachexia and these inflammatory cytokines may drive myocyte cell death (19).

Mechanistically, there are a number of gut bacteria that have been singled out as gut barrier function disabling and inflammatory promoting. In lung cancer patients with cachexia, while gut α-diversity was not significantly perturbed when compared to patients without cachexia, a few bacteria species were significantly different. For example, a lower abundance of *Prevotella copri* was observed in patients with cachexia and this correlated with reduced plasma levels of postulated myogenic branched chain amino acids (BCAAs) isoleucine and leucine. Lower levels of *Faecalibacterium prausnitzii*, a gut bacterium with known anti-inflammatory effects (20), was also observed in cancer patients with cachexia and this may tip the balance to a more proinflammatory state in patients with cachexia. Inversely significantly higher levels of *Klebsiella oxytoca*, a bacterium associated with reduced gut barrier function (21), was seen in lung cancer patients with cachexia, and together with reduced gut barrier function, bacterial translocation and inflammatory cytokines may drive cachexia in patients with cancer (22).

## Underpinning mechanisms for gut microbiome and muscle crosstalk

Most published mechanistic studies leverage on the pathophysiology of small animals. Altered patterns of microvillus formation and reduced cell renewal were observed in mice depleted of gut microbiota. Since microvilli are involved in absorption of both macro- and micro-nutrients, pathologies affecting the microvilli may impact overall metabolism including muscle mass and function (23, 24). In a study, GF mice had low levels of 25-hydroxyvitamin D (25D), 24,25-dihydroxyvitamin D (24,25D) and 1,25-dihydroxyvitamin D, and were hypocalcaemic. After 8 commensal bacteria were introduced, the levels of 25D and 24,25D increased to the same extent as conventionalisation. Fibroblast growth factor (FGF)23 was initially high in GF mice, but eventually reduced and normalized the vitamin D and calcium levels (25). GF mice also exhibited increased bone mass due to reduced number of osteoclast per bone surface, and it normalized with colonization by normal gut microbiota (26). However, associations between alterations in gut microbiota and changes in muscle function could also be mediated by gut-derived metabolites such as SCFAs, which play an important role in modulating lipid, carbohydrate and protein metabolism in skeletal muscle. Although SCFAs are formed in the gut, effective concentrations can be found circulating in the body (27). SCFAs are formed from the fermentation of fibers such as non-digestible carbohydrates, and they include acetate, propionate, and butyrate. These SCFAs are critical for maintaining the integrity of the epithelial barrier, the loss of which compromises barrier permeability and increases the risk of bacteria or bacterial antigen translocation. This in turn triggers the inflammatory cascade which may underpin chronic inflammation observed in obesity and insulin resistance (28).

Butyrate, of which higher levels are found in older adults with normal compared to low muscle mass, has been shown to promote mitochondria biogenesis (29, 30). When female mice were given a dietary supplement containing butyrate throughout the gestation and lactation phases, mitochondrial biogenesis was correspondingly enhanced in the offspring, evident by increased ATP content, mitochondrial DNA-encoded gene expression and uncoupling protein 3 (UCP3) in the gastrocnemius muscle of the offsprings (30). Separately, high functioning sedentary older adults had higher levels of *Barnesiella* and *Prevotella* genera, including the species *Barnesiella intestinihominis*, as compared to their low functioning sedentary counterparts. Notably, *Barnesiella* and *Prevotellaceae* were shown to be gut producers of SCFA (31, 32). Among older persons with low functional muscle strength, those with higher levels of SCFAs correlated with greater muscle strength, suggesting that SCFAs may contribute to the observed enhanced muscle strength (33).

When circulating SCFAs were significantly reduced in plasma of antibiotic treated mice, exercise endurance in these mice correspondingly dropped, which was again restored with acetate infusion. Caecal acetate, propionate, and butyrate were eliminated in antibiotic treated mice, suggesting that gut microbe derived SCFAs, especially acetate, may be an important energy substrate during endurance exercise (34). Besides its effects on mitochondria, SCFAs also affects muscle health by altering nuclear gene expression.

Administration of dexamethasone to C2C12 myotubes resulted in increased Atrogin-1 expression. This effect on *Atrogin-1* expression was reduced when the C2C12 myotubes were treated with a cocktail of SCFAs, similar to those generated from fermentation of dietary polysaccharides. In addition, treatment of GF mice reduced the expression of *Atrogin-1* in the tibialis anterior and increased the expression of *MyoD* (2).

## Diversified effects of microbial biomolecules for the muscle

Indoxyl sulfate is a gut microbiome derived uremic toxin and is known for its pro-inflammatory properties in chronic kidney disease (CKD) patients (35). Administration of indoxyl sulfate was observed to reduce muscle mass in mice. It significantly increased intracellular ROS production in C2C12 myoblast cells, which plays an important role for skeletal muscle atrophy through various mechanisms (36). Also, indoxyl sulfate caused an increase in expression of myostatin (*Mstn*) and *Atrogin-1* mRNA through the arylhydrocarbon receptor (AHR) pathway, inhibiting cell proliferation and myotube formation (37). Another way in which gut microbiome could negatively affect muscle health is through LPS. LPS is potent endotoxin present in the outer membrane of Gram negative bacteria and is known for its pro-inflammatory properties. Disrupted intestinal barrier may cause translocation of these bacterial components into systemic circulation which may in turn result in inflammation, *via* the Toll-like Receptor 4 (TLR4) pathway, resulting in muscle atrophy (38, 39). This is also seen in chronic diseases where pro-inflammatory factors appear to be the unifying factor of muscle atrophy (40). It was found that LPS decreased the formation of multi-nucleated myotubes and inhibited myogenic differentiation *in vitro* (41) suggesting that changes in gut permeability may allow leakage of bacterial derived glyco-peptides into the circulation which then affects the function of distal tissues such as skeletal muscle.

## Muscle function in common gastrointestinal diseases

Diseases of the gastrointestinal tract such as inflammatory bowel diseases (IBD) and celiac disease (CD) are associated with a decline in muscle function and cachexia (42). Studies have shown that the gut microbiota in IBD patients were significantly altered from that of healthy individuals, and that dysbiosis of gut microbiota accompanied by disruption of diet-microbe interactions, results in damage to intestinal microbial barrier. For instance, in Crohn's Disease, over 50% of patients presented with adherent-invasive *E. coli* colonization in intestinal mucosa (43). Dysbiosis, defined as a disease associated imbalance in the gut microbial community, was reported for IBD. Decreased *Firmicutes* and *Bacteroides*, and increased *Enterobacteriaceae*, were observed in IBD. This microbe diversity shift disrupted the intestinal barrier integrity through increased abundance of mucolytic bacteria facilitating increased penetration of pathogens into intestinal tissue (44). Furthermore, it has been reported that more than a third of IBD patients suffer from sarcopenia. Similar to what was observed in the frail population, there was a reduction in *F. prausnitzii*, a SCFA producer with significant anti-inflammatory function (45). This raises the possibility that the decrease in anti-inflammatory gut microbiota with disruption of the epithelial barrier function in IBD may trigger the inflammatory cascade with release of proinflammatory cytokines such as TNF-α and IL-6 affecting muscle mass and function.

## Exercise as a potential modulator of intestinal microbiome composition

While physical exercise directly benefits muscle function, either anatomically through maintenance of muscle sarcomere density or metabolically through increase of myocyte energetics, it has also been suggested have indirect benefits on the muscle through for example the modulation of gut commensals. Physical activity performed continuously at low doses can increase the abundance of health promoting gut bacteria such as *Bifidobacterium spp, R. hominis, A. muciniphilia* and *F. parusnitzii* (46). However, the relationship between gut microbiota and muscle health remains complex. In addition to diet, exercise is a positive modulator of gut microbiota biodiversity and this has been reviewed extensively (47, 48). Conversely, frailty, as determined using the Rockwood Frailty Index, had a negative correlation with gut microbiota α-diversity (49). The microbiota, especially those that produce metabolites such as SCFA, are important to endurance athletes, because they can supply around 10% of the energy needed by the host (50). With regular physical activity, muscle fibers release myokines such as IL-6, contributing to an overall systemic anti-inflammatory tone (51, 52). In turn, this may help to protect the microbiota from changes caused by inflammatory conditions such as IBD and T2DM (53). High-fat diet fed (i.e., pre-diabetic) mice which received fecal microbiota transplantation (FMT) from actively exercising mice showed improved metabolic parameters such as insulin sensitivity, suggesting that microbes obtained from a physically active host contributes positively to overall metabolic function (54).

While exercise is associated with numerous health benefits, intense exercise can result in acutely increased gut permeability, and reduced mucus production, allowing pathogens such as LPS in ultra-endurance runners to enter the bloodstream and causing inflammation (55, 56). Exercise-induced gut barrier disruption is observed with an acute rise in inflammatory markers, such as plasma TNF-α (57). These changes were however found to be reversible and thus may not outweigh the benefits of exercise. Separately, in a 6-week intervention study amongst older adult males who participated in twice weekly resistance training, resistance training did not alter much of their gut microbiome composition (58). Although a subsequent *in silico* analysis revealed a paradoxical increase in mucin synthesis, the study stopped short of validating changes to bacterial translocation and systemic inflammation (47). Taken together, more studies with prospective follow-up are required to better understand the longitudinal impact of these cross-sectional associations of exercise and nutrition with gut microbes and systemic inflammation. These studies also point to the judicious use of antibiotics because inappropriate or excessive use of broad-spectrum antibiotics is a major iatrogenic contributor to a deranged gut microbiome.

## Other forms of interventions involving the gut microbiome

Besides physical exercise, alterations either through microbiome depletion/ reconstitution, FMT, diet interventions or pre-/probiotics supplementation may offer a new approach to address the problem of frailty by targeting the gut-muscle axis (59). Probiotics refer to defined viable microorganisms, sufficient amounts of which reach the intestine in an active state and thus exert positive health effects. Prebiotics, on the other hand, refer to selectively fermented ingredients that allow specific changes, both in composition and/or activity in the gastrointestinal microflora that confers benefits upon host wellbeing and health, such as non-digestible oligosaccharides (60).

Since increased gut permeability is seen in cachexic mice and patients, supplementation with probiotics may restore gut barrier dysfunction thereby lowering pathogen leakage and systemic inflammation. Supplementation with *Lactobacillus* and *Bifidobacillus* has the potential to reduce age-induced and cancer induced muscle loss, while supplementation with lactobacillus is suggested to ameliorate muscle wasting *via* increasing butyrate production and decreasing gut permeability.

Most recently, two studies have revealed interesting insights. The SAMP8 mouse is commonly used as a pre-aging animal model because it starts to display an aging phenotype from 4 months of age. Probiotic supplementation of *Lactobacillus casei Shirota* (1 × 10^8^ or 1 × 10^9^ CFU/mouse/day by oral gavage) decreased the senescent scores and increased muscle mass in SAMP8 mice. Furthermore, it helped to maintain muscle strength in the aged mice, as seen from the higher grip force. It also reduced age related increases in inflammation by down regulating the proinflammatory cytokine TNF- α and upregulating the anti-inflammatory cytokine IL-10. In contrast to the fall in SCFAs usually seen in aging, *Lactobacillus casei Shirota* helped to maintain the butyrate levels in the aged mice (61). This study involved a small case series of six non-agenarian older adults (mean age: 90.8 ± 5.4 years) with sarcopenia who were administered the prebiotic 1-kestose (10 g/day for 2 weeks), there was an increase in the intestinal *Bifidobacterium longum* population along with increased skeletal muscle mass index and reduced body fat percentage (62). This study provided proof-of-concept evidence regarding the potential clinical benefit of prebiotic supplementation even in the oldest-old age group. Although treatment with prebiotics and probiotics may be promising in improving the gut microbiota (63), there are too limited studies at the moment to associate, let alone validate, whether its gut microbe effect carries on to muscle health amongst people with frailty syndrome.

## Conclusion

In this perspective, we start with a brief overview on how gut microbiota can influence muscle health through various mechanisms and on how various microbes can be altered in certain muscle disease states. We discuss recent experimental and clinical evidence in support of microbiome impacting muscle mass, with an overall consensus that gut microbes impact muscle mass, either positively or negatively, depending on the microbe strain. This is supported by evidence that microbiome manipulation through either FMT or antibiotic administration can reverse phenotypes in GF and SPF mice, respectively. Meanwhile, human studies are beginning to show that microbiome composition is associated with muscle mass and function, paralleling changes in inflammatory markers in patients with frailty and other cachexic conditions. We looked into as many relevant papers as possible without bias or application of any exclusion criteria (i.e., not a systemic review) when gathering evidence for this perspective, but in doing so may have inadvertently missed a few relevant papers. This remains a limitation of this piece. Nevertheless, while much remains unknown about how microbiome interacts with muscle, this emerging field of research holds promise for improving our understanding of sarcopenia and other age-related muscle loss. Information on human, and animal, gut-muscle axis are now compiled into a single table (Table 1). Importantly, clinical studies will be needed to determine whether microbiome modulation *via* diet modification or pre/probiotic supplements can improve muscle health in humans. With continued research, we may 1 day be able to use microbiome manipulation to combat sarcopenia and other disorders of muscle loss.

**Table 1:** List of human and animal studies that relate gut microbes to muscle and its related phenotype.

**Study context**	**Muscle and related phenotype**	**Gut microbiota**	**References**
**Human studies**			
Loss of community associated microbiota in long stay subjects correlated with increased frailty.	Significant associations with Barthel Index and functional independence measure (FIM).	Long stay care environment subjects had high proportion of *Bacteroidetes*, whereas individuals living in the community dwelling had a high level of *Firmicutes*. Long stay care subjects had significantly less diverse microbiota with less SCFA-producing bacteria compared to community and rehabilitation subjects.	(64)
Gut microbe changes in patients with sarcopenia compared to age-matched healthy cohort.	Grip strength < 28 kg (male) or < 18 kg (female).	Ruminococcus positively correlated with grip strength in sarcopenic cohort.	(65)
Alterations in gut microbiome after weight loss in human subjects	No measure of muscle mass other than correlations of general adiposity.	High levels of *Firmicutes* in obese subjects. Low levels of *Bacteroidetes* in obese individuals partially normalized with weight loss.	(66)
Obese monozygotic and dizygotic twins have reduced phylogenetic diversity.	No measure of muscle mass.	Lower proportion of *Bacteroidetes and i*ncreased abundance of *Actinobacteria* while the levels of *Firmicutes* remained unaltered.	(67)
Type-2 diabetes mellitus	Reduced muscle insulin sensitivity.	Proportions of the *Firmicutes*, and specifically the *Clostridia* class, were reduced, while the *Bacteroidetes* and the class *Betaproteobacteria* were enriched in a group with T2DM compared with controls.	(68)
		Moderate degree of gut dysbiosis, characterized by an increase in certain opportunistic pathogens, such as number of *Clostridium* spp. in addition to important gut microbes including *Akkermansia muciniphilia, Bacteroides* spp. and *Desulfovibrio* spp.	(69)
Frailty in older adults was associated with reduced gut microbiota diversity.	Measures of frailty index (FI) including Rockwood Frailty Index.	*Enterobacteriaceae* were increased, whereas *Bacteroides/Prevotella* and the bacterial species *Faecalibacterium prausnitzii* sharply declined.	(12, 49)
Chronic liver disease patients with low skeletal muscle mass had lower branch chain amino acid (BCAA) synthesis genes, by 16S RNA, compared to chronic liver disease patients with normal skeletal muscle mass.	Skeletal muscle.	Lower Firmicutes/ Bacteroidetes ratio. *Coprobacillus, Catenibacterium and Clostridium* were also lower while the *Bacteroides* was higher. Microbiome characterized by high relative abundance of gram negative bacteria with LPS	(10)
In patients with advanced gastric cancer, cachexic patients had a higher prevalence of bacterial translocation than non-cachexic patients.	Cachexia measured as weight loss of >10% of the pre-illness state.	Bacterial DNA detected from the portal vein indicative of reduced intestinal permeability and increased bacterial translocation in subset of cachexia patients. Higher alteration of intestinal flora was noted in cachexic patients.	(19)
Cachectic lung cancer patients	Loss in overall muscle mass.	*Prevotella copri* showed significantly lower abundance in cachectic patients. *Klebsiella oxytoca* is significantly higher in lung cancer patients with cachexia *Fecalibacterium prausnitzii* is significantly more abundant in non-cachectic patients. Significant enrichment of microbiota LPS biosynthesis pathway.	(22)
Elderly older than 65 years old with low muscle mass	Skeletal muscle (based on body composition, grip strength, gait speed and flexibility).	*Firmicutes/Bacteroidetes* ratio was significantly reduced in the low muscle mass group. *Marvinbryantia* spp. (SCFA producer) was decreased significantly, *Flavonifractor* spp. (flavonoid degrader) was enriched and Fecal butyrate was significantly diminished and correlated with skeletal muscle mass index	(29)
Muscle impairment in older adults	Physical frailty, based Short Physical Performance Battery (SPPB), low appendicular muscle mass (aLM), and absence of mobility disability (i.e., ability to complete the 400-m walk test).	Increase in *Oscillospira* and *Ruminococcus* and decrease in *Barnesiellaceae* and *Christensenellaceae* in physically frail subjects.	(70, 71)
	Sedentary women subjected to exercise interventions including aerobic exercise training (brisk-walking).	Relative abundance of Bacteroides significantly increased in sedentary women after 12 weeks of aerobic exercise. Abundance of Bacteroides positively correlated with an increased physical performance assessed by the 6 min walking test	
Chronic alcohol consumption had a loss of muscle strength	Handgrip strength was significantly lower in the alcohol overconsumers group compared to control patient group.	Higher relative abundance of *Proteobacteria, Sutterella, Clostridium* and *Holdemania*. Lower relative abundance of *Faecalibacterium* with reduced fecal SCFAs levels	(72)
Difference in gut microbiota profile between women with active lifestyle and sedentary women.	Sedentary women performed < 3 days of exercise per week for 30 min at moderate intensity.	Higher abundance of health promoting bacterial species in active women, including *Faecalibacterium prausnitzii, Roseburia hominis* (butyrate producers), Bifidobacterium spp and *Akkermansia muciniphila*.	(46)
	Active women performed at least 3 h of physical exercise per week.	Lower Bacteroidetes in the active group.	
In a 6 week endurance exercise study without dietary changes amongst previously sedentary overweight women, metagenomic analysis revealed taxonomic shifts.	Non-significant increase in *M. vastus lateralis* thickness	Increase in *Dorea, Anaerofilum and Akkermansia*. Decrease in *Porphyromonadaceae, Odoribacter, Desulfovibrionaceae and Enterobacteriaceae*	(73)
Athletes vs. normal individuals	Overall impact of exercise on gut microbiome.	Higher levels of SCFAs (acetate, propionate, butyrate and valerate) in athletes relative to controls. Concentrations of propionate strongly correlated with protein intake. Concentration of butyrate was shown to have a strong association with the intake of dietary fiber.	(74)
In male runners, multistrain probiotic supplementation significantly increased running time to fatigue. In addition, probiotic supplementation lead to small to moderate reduction in intestinal permeability and gastrointestinal discomfort.	Run time to fatigue.	*Probiotic supplementation consisting of Lactobacillus acidophilus, Lactobacillus rhamnosus, Lactobacillus casei, Lactobacillus plantarum, Lactobacillus fermentum, Bifidobacterium lactis, Bifidobacterium breve, Bifidobacterium bifidum*, and *Streptococcus thermophilus*	(57)
High performing martial arts athletes have significantly different gut microbial richness and diversity as compared to the lower-level martial arts athletes	Elite atheletes.	Genera *Parabacteroides, Phascolarctobacterium, Oscillibacter* and *Bilophila* were enriched in the higher-level athletes, whereas *Megasphaera* was abundant in the lower-level athletes. The abundance of the genus *Parabacteroides* was positively correlated with the amount of time participants exercised during an average week.	(75)
Athletes had relative increases in pathways (e.g., amino acid and antibiotic biosynthesis and carbohydrate metabolism) and fecal metabolites (e.g., microbial produced SCFAs acetate, propionate and butyrate) associated with enhanced muscle turnover and overall health when compared to control groups.	Althetes vs. control.	Higher levels of microbial derived SCFAs	(17)
Frailty associated changes in gut microbiota amongst community dwelling adults.	Confirmed with Fried et al. definition for frailty.	As compared to controls, the frailty groups had higher *Akkermansia, Parabacteroides, Klebsiella* and lower *Faecalibacterium, Prevotella, Roseburia, Megamonas, Blautia*.	(76)
Intensive, prolonged exercise causes gut dysbiosis in female endurance runners	15 female Japanese elite runners with mean monthly running distance of 547 km.	The abundance of Deferribacteres was significantly higher in the endurance runner group. Concentration of succinate (an undesirab gut bacteria metabolite) is significantly higher in female endurance runners.	(55)
6 weeks of resistance training of older adult males	DXA whole and lower body lean mass and leg extensor peak torque increased.	Increase in mucin biosynthesis. No change in microbiome diversity, LPS levels, SCFA, and mucin degradation	(58)
High fiber diet may be involved in mechanisms related to whole body lean mass and physical functioning in older adults	Physical function measured by short physical performance battery and grip strength.	Higher levels of *Ruminococcus, Lachnospira, and Clostridia*, and genes related to butyrate and SCFA production	(77)
Prebiotic 1-ketoase resulted in recovery of muscle atrophy in super elderly patients with sarcopenia	Increased total muscle mass, trunk mass, skeletal muscle index, skeletal muscle mass increased, as measured using multi frequency bioimpedance analysis device.	Increased *Bifidobacterium longum* in intestine after 1-ketose administration for 12 weeks	(62)
Prebiotic administration improved frailty criteria amongst elderly	Improvement in self-reported exhaustion score and better handgrip strength in the dominant hand.	Mixture of inulin and fructoligosaccharide intake over 13 weeks	(78)
**Animal studies**			
Genetically obese (ob/ob) mice have altered gut microbiome when compared to lean (ob/+) littermates.	High fat to lean mass ratio.	Higher proportions of *Firmicutes* and lower levels of *Bacteroidetes*	(79)
Fewer but larger muscle fibers compared to germ free (GF) mice.	Tibialis anterior (Fast oxidative muscle).	GF mice with conventionalisation.	(2)
Increased expression of FoxO3, *Atrogin-1* and *Murf-1* encoding E3 ubiquitin ligases, which are known to be involved in muscle atrophy in GF mice, reduced with transplantation of gut microbes.	Similar trend also observed in soleus (slow oxidative muscle) and extensor digitorium longus (fast glycolytic muscle).	GF mice with conventionalisation.	(2)
Reduced expression of myosin heavy genes and glycolytic genes, restored with transplantation of gut microbes.	Quadriceps (fast glycolytic) muscle.	GF mice with conventionalisation.	(2)
Slower growth rates and transplant restored growth in later days.	Reduced growth of lean mass.	GF piglet with conventionalisation.	(3)
Lower total SCFAs content in the colon Muscle mass smaller and muscle fibers thinner	Longissimus dorsi muscle	GF piglet with conventionalisation.	(3)
Increased muscle mass, grip strength, and endurance swimming time	Increased overall muscle mass and function.	Long term *lactobacillus plantarum* TWK10 supplementation in mice	(80)
Obese mice treated with prebiotic, oligofructose.	Increase in lean and skeletal muscle mass.	Associated with increases in family-level *Prevotellaceae*, and genus-level *Prevotella and Barnesiella*	(81)
Mouse model of leukemia (transplantation of BaF3 cells containing ectopic expression of Bcr-Abl), treated with probiotic supplements.	Decreased muscle atrophy gene expression (i.e., Atrogin-1, MuRF1, LC3, Cathepsin L), and increased muscle mass.	Lactobacillus spp. led gut dysbiosis was treated with *Lactobacillus reuteri* and *Lactobacillus gasseri* supplementation.	(13)
Microbiota from high functioning (HF) older adults transplanted into GF mice led to increased muscle strength.	Forelimb grip strength.	Genus level *Paraprevotella, Akkermansia, Barnesiella, Eubacterium, Prevotella* and *Coprobacillus* were higher in the HF-mice compared to LF-mice one-month after transplantation.	(6)
Antibiotic induced dysbiosis of the gut microbiome blunted hypertrophic response of type 1 fibers. It does not impair skeletal muscle fiber type shift in response to training	Soleus muscle.	Antibiotic (metronidazole, neomycin, ampicillin, vancomycin, streptomycin) treated mice had reduced microbiota composition and resulted in enlarged caecum.	(5)
Antibiotics blunted hypertrophy, myonuclei accretion, satellite cell abundance and fiber-type shift to type 2a fibers.	Plantaris muscle.		(5)
*Klebsiella oxytoca* is increased in tumor bearing mice with cachexia independently of anorexia.	Loss in overall muscle mass.	*Klebsiella oxytoca*	(21)
GF mice has increased bone mass	GF mice have decreased frequency of CD4+ T cells and CD11b+/GR 1 oesteoclast precursor cells in bone marrow.	Conventionalisation of GF mice normalizes bone mass.	(26)
Maternal butyrate supplementation throughout gestation and lactation did not affect offspring weight but Type 1 myosin heavy chain, mitochondria transcription factor A, PPAR-coactivator-1a and uncoupling protein 3 (UCP3) increased in the gastrocnemius muscle of rats.	Gastrocnemius.	Implications on butyrate-producers in maternal gut.	(30)
Low gut-derived acetate leads to reduced exercise endurance.	Overall impact on exercise endurance in mice.	2 weeks antibiotic treatment reduced exercise endurance. In the cecum, acetate, propionate and butyrate became almost undetectable. Antibiotic treatment associated with a larger population of *Firmicutes* and a smaller proportion of *Bacteroidetes*. Exercise capacity was restored by continuous acetate infusion (but not by butyrate infusion), suggesting that plasma acetate may be an important source of substrate during endurance exercise.	(34)
		6 week low microbiome-accessible carbohydrate (LMC) significantly reduced exercise capacity, and fecal and plasma SCFA concentrations. Ratio of Firmicutes: Bacteroidetes was also higher.	(34)
		In LMC-fed group, there were more *Lactococcus* and *Allobaculum* and lower *Prevotella* (which generates SCFA) and S24-7. Low dietary fermentable fiber content alters the composition of the microbiome in favor of bacteria that produces less SCFA	(34)
In hemodialysis patients, serum levels of indoxyl sulfate or p-cresol suphate may be reduced by either pre-biotics or pro-biotics administration.	Indoxyl sulfate inhibits myotube formation and increases factors related to skeletal muscle breakdown. P-cresol negatively affects the vascular endothelium.	Pre-biotics (oligofructose enriched inulin). Pro-biotics (*Bifidobacterium longum*).	(36, 82, 83)
Disruption of intestinal barrier leads to the development of metabolic disease.	Reduced muscle insulin sensitivity.	Bacterial lipopolysaccharides in systemic circulation.	(84)
High fat diet induces change in the composition of gut microbiota	Implied alteration to fat: lean mass ratio.	Reduction in *Bifidobacterium* spp. and *Eubacterium rectale*–*Clostridium coccoides* (Gram-positive bacteria) as well as *Bacteroides* (Gram-negative bacteria).	(85)
		Negative correlation between Bifidobacterium spp. and plasma LPS levels has been observed, and an increase in bifidobacterial induced by prebiotic intake reduces endotoxaemia.	(85)
		Decrease in Bacteroidetes and an increase in Firmicutes	(85)
Colonization of GF mice with fecal samples from age matched conventionally leads to reduction in whole body lean mass by 7–9%, with a 57% increase in total body fat content.	Reduced lean mass.	Conventionalisation results in elevations in liver mRNAs encoding two key enzymes in *de novo* fatty acid synthesis pathway. It also results in microbial suppression of intestinal fasting-induced adiposcyte factor (Fiaf) which promotes adiposity.	(7)
Exercise changes microbiota composition and increases n-butyrate concentration in the rat cecum.	Voluntary wheel running.	Increased n-butyrate, with no change in SCFA concentrations.	(86)
Diet exerted more influence than exercise in shaping the gut microbiota. The beneficial effects of diet and exercise are transmissible *via* FMT. HFD mice receiving FMT from normal diet exercised donor mice had reduced weight and improved whole-body metabolic profiles.	Fat weight.	Transmissible effect of FMT were associated with bacterial genera *Helicobatcer, Odoribacter* and AF12 and overexpression of oxidative phosphorylation and glycolysis genes. FMT has comparable effect to exercise in reducing body and fat weight in mice fed with high fat diet.	(54)
		As *Odoribacter* is a known producer of SCFA, such as acetate, propionate and butyrate, increased *Odoribacter* may contribute to decreased inflammation,	(87)
Differences in gut micriobiota of GF mice after FMT from children donors of different nutritional status	Nutritional status assessed based on weight-for-height Z-Score (WHZ).	*Faecalibacterium prausnitzii* were predominant in higher muscle mass recipient mice donated by healthy infants, while *Clostridium neonatale* were predominant in recipients donated by malnourished and underweight infants.	(88)
Antibiotic administration increased the myofiber density and expression of genes related to type I and type IIb myofibers in longissimus muscle	Longissimus muscle.	Antibiotic treatent, decreased *Ternericutes, Dialister, Asteroleplasma*, Prevotella, Campylobacter, Selenomonas, Misuokella, Acidaminococcus, and increases *Firmicutes, Bacteroidetes, Phascolarctobacterium, Paraprevotella, Oscillibacter, Coprococcus, Blautia, Ruminococcus*.	(9)
Probiotics (*Fecalibacterium prausnitzi*) increased muscle mass in mice	Gastrocnemius muscle harvested and weighed.	Oral *F*. prausnitzii increased muscle mass which could be due to enhanced mitochondrial respiration, improved insulin sensitivity, modified gut microbiota composition with increased abundance of *Lactobacillus* and *Streptococcus*, and improved intestinal integrity.	(89)
Probiotic delays the appearance of senescence and age-related muscle mass deposition in SAMP8 mouse, and age-related decline in muscle strength	Muscle strength evaluated using the four-limb hanging and grip strength tests.	Administration of *Lactobacillus casei* Shirota by oral gavage for 12 weeks.	(61)
Pulsed antibiotic treatment (PAT) (either 1. Tylosin or 2. Amoxicillin or 3. Mixed) produced mice with larger bones and higher lean mass than control. It trended toward increased fat in tylosin-treated mice and bone in amoxicillin-treated mice.	Lean mass.	PAT decreased richness and Shannon eveness after one antibiotic pulse. Bacteroidetes in the mixed group and some on tylosin were dramtically reduced. It was relatively unchanged in the amoxicillin mice.	(8)
		On high fat diet (HFD), members of the phylum Firmicutes increased at the expense of Bacteroidetes in untreated mice. Many similar families were changed, but the changes were not significant in amoxycillin-treated mice on HFD. In tylosin mice on HFD, changes were partially in the same direction (Streptococcaceae, Clostridiales other, Firmicutes other and Prevotellaceae) and partially in the opposite direction (Erysipelotrichaceae, Ruminococcaceae, Rikenellaceae, Bacteroidales other and Bacteroidetes other) compared to untreated HFD mice.	(8)

## Data availability statement

The original contributions presented in the study are included in the article/supplementary material, further inquiries can be directed to the corresponding author.

## Author contributions

WC, WL, SW, and YA wrote the manuscript. YL, EC, GF, and YA edited the manuscript. All authors contributed to the article and approved the submitted version.
